# Ocular Surface Disease Index and Ocular Thermography in Keratoconus Patients

**DOI:** 10.1155/2020/1571283

**Published:** 2020-03-08

**Authors:** Orsolya Németh, Achim Langenbucher, Timo Eppig, Sabine Lepper, Georgia Milioti, Aladin Abdin, Zoltán Zsolt Nagy, Berthold Seitz, Nóra Szentmáry

**Affiliations:** ^1^Department of Ophthalmology, Saarland University Medical Center, Homburg, Saarland 66424, Germany; ^2^Department of Ophthalmology, Markusovszky University Teaching Hospital, Szombathely 9700, Hungary; ^3^Department of Ophthalmology, Semmelweis University, Budapest 1085, Hungary; ^4^Experimental Ophthalmology, Saarland University, Homburg, Saarland 66424, Germany

## Abstract

**Purpose:**

Keratoconus (KC) has been defined as a “noninflammatory” corneal disease, but recent studies have noted a potential inflammatory origin. We analysed the Ocular Surface Disease Index (OSDI) and ocular surface temperature (OST) in KC patients compared to controls. *Patients and Methods*. A total of 179 eyes in 90 patients with KC (topographic keratoconus classification 0-1 to 4, age 36.1 ± 12.5 years, 65.9% males) and 82 eyes in 41 controls (age 36.4 ± 12.8 years, 47.6% males) were examined. The participants completed the OSDI questionnaire and underwent corneal topography, tomography, and thermography. Additional outcome measures were vision- and discomfort-related OSDI subscores and mean OST  at the corneal centre during 10 seconds of sustained eye opening after blinking.

**Results:**

The OSDI score (31.4 ± 22.4 vs. 17.5 ± 17.9) and vision- (17.7 ± 14.6 vs. 10.5 ± 13.2) and discomfort-related (14.3 ± 10.7 vs. 9.4 ± 10.5) OSDI subscores were significantly higher in KC patients than in controls (*p* < 0.001). We found no significant difference in the central corneal OST (34.2 ± 0.6°C vs. 34.2 ± 0.7°C; *p* < 0.001). We found no significant difference in the central corneal OST (34.2 ± 0.6°C vs. 34.2 ± 0.7°C; *p* < 0.001). We found no significant difference in the central corneal OST (34.2 ± 0.6°C vs. 34.2 ± 0.7°C; *r* > 0.174, *p* < 0.001). We found no significant difference in the central corneal OST (34.2 ± 0.6°C vs. 34.2 ± 0.7°C; *r* > 0.174, *r* > 0.174,

**Conclusion:**

KC patients had increased OSDI scores and vision- and discomfort-related OSDI subscores without an increase in the OST compared to a normal population. OSDI score/subscores weakly correlate with SAI and SRI but do not correlate with OST in KC patients or controls. Vision- and discomfort-related symptoms of KC have to be managed in parallel in ophthalmological practice, but the necessity of anti-inflammatory treatment cannot be verified through ocular thermography.

## 1. Introduction

Thermography is used in many fields of medicine, including angiology [1], oncology [2], and rheumatology [3]. Since Mapstone [4–6] introduced infrared thermography of the ocular surface, the method has become widely used. In ophthalmology, ocular surface temperature (OST) has been investigated in ocular inflammation [7], tear film abnormalities [8], analysis of bleb function after glaucoma surgery [9] and after corneal refractive surgery [10] and cataract surgery [11], and in the evaluation of ocular blood flow [12].

OST may be influenced by environmental and ocular factors. In the environment, changes in the ambient temperature may influence OST, but it reaches a plateau of 36°C at an ambient temperature of 40°C [13, 14]. Wind, air conditioning, or any kind of air flow affects OST through increased evaporation [15], and lower air humidity increases lacrimal evaporation [16]. Blinking interrupts corneal exposure to the environment and redistributes the tear film and its temperature [17].

OST also depends on ocular factors, such as the quality and quantity of the tear film and heat conduction and convention of the aqueous humour, which is determined mainly through blood flow in the ciliary body and through retrobulbar haemodynamics [18]. As the cornea is an avascular tissue, the central corneal temperature is mainly influenced by tear film evaporation and heat convection and conduction of the aqueous humour [18]. Nevertheless, the temperature of the peripheral cornea may also be influenced by blood flow in the perilimbal vessels [12].

Keratoconus (KC) is a bilateral, asymmetric condition characterized by progressive thinning and deformation of the corneal tissue, which may lead to significant visual impairment due to irregular corneal astigmatism [19]. Its prevalence is approximately 1 : 2000 in the Caucasian population, but its exact aetiology remains unknown. Although KC cases are sporadic, some studies have reported autosomal dominant or recessive inheritance [20]. Eye rubbing may be the most important environmental factor related to the development of KC. Therefore, patients with a history of atopy may have a higher risk of developing KC [21].

Although KC is defined as a “noninflammatory” corneal disease, several studies have reported a potential inflammatory origin. For example, proinflammatory cytokines IL-6, IL-1*β*, IFN-*γ*, and TNF-*α* have been demonstrated in the tear film of KC patients [22–24]. In clinical studies on KC patients, ocular surface disease is characterized by worse tear quality, significantly lower break-up time (BUT), and higher fluorescein and rose bengal staining scores than the normal population [25]. A correlation between ocular surface disease and KC stage has also been verified [25]. Although some of the KC screening indices are also sensitive to dry eye syndrome [26], no interaction between measures of dry eye syndrome and topographic/tomographic changes in KC patients could be shown [27].

To add insight into the relationship between ocular surface disease and KC, we analysed the Ocular Surface Disease Index (OSDI) and OST in KC patients compared to controls.

## 2. Patients and Methods

All examinations were performed following the regulations of the Declaration of Helsinki. Our study was approved by the Ethics Committee of Saarland/Germany (no. 41/18), and informed consent was obtained from all participants at the Homburg Keratoconus Center [28].

Patients with the diagnosis of KC who had not had previous ocular surgery were included in the study. Additional exclusion criteria were tight palpebral fissure, rapid eye movements during the examination, diagnosis of pellucide marginal degeneration, and keratoglobus. KC was diagnosed by slitlamp examination and corneal topography (TMS-5, Tomey, Erlangen-Tennenlohe, Germany) and tomography (Pentacam, Wetzlar, Germany). Previous contact lenses were not an exclusion criterion, but patients had to be examined after at least 2 days without wearing contact lenses.

Each participant completed the Ocular Surface Disease Index (OSDI) questionnaire. A total OSDI score, vision-related subscore (derived from questions about vision and task performance), and discomfort-related subscore (derived from questions about ocular surface discomfort) were calculated for each participant as described by Mathews et al. [29]. Score ranges were designated as normal (0–12), mild (13–22), moderate (23–32), or severe (33–100) ocular surface disease.

As the patients filled out the OSDI questionnaire, the OST  adapted to the ambient temperature of the standardized examination room. We then measured the OST using the TG-1000 Ocular Surface Thermographer (Tomey, Erlangen-Tennenlohe, Germany). All examinations were performed by the same examiner to eliminate interexaminer variation [30]. During the measurements, a standard environment was maintained in the examination room with an average room temperature of 23.9 ± 1.6°C and humidity of 32.4 ± 6.7%. The doors and windows in the examination room were closed to minimize air flow, which has been reported to have a significant effect on the OST [15].

**Figure 1:**
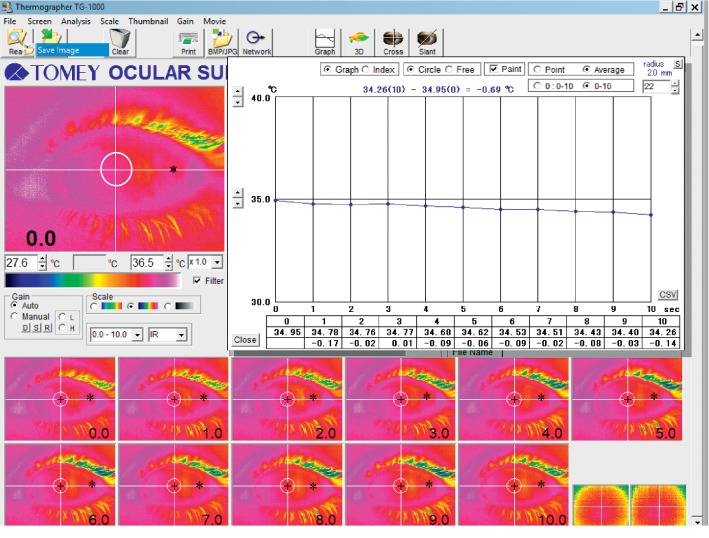
Ocular surface thermography in a keratoconus patient. We extracted mean ocular surface temperature values from the corneal centre (see x) and 2 mm from the corneal centre nasally, temporally, superiorly, and inferiorly during 10 seconds of sustained eye opening after blinking (crossing of the white circle and the white lines). Data on ocular surface temperature conjunctivally, 8 mm temporally, from the corneal centre (^*∗*^) were also collected.

The OST was measured using the method described by Mori and associates: the participants blinked normally, closed both eyes for 5 seconds, and then kept their eyes open for more than 10 seconds [31]. During the examination, the participants' head was placed in a standard ophthalmic chin and head rest, and they were instructed to look straight ahead. For each measurement, a sequence of 11 OST images was taken from baseline to 10 seconds after eye opening (1 per second). With a lateral resolution of 70 *μ*m in both the horizontal and vertical directions, 320 × 240 data points were stored with each image. We extracted mean OST values at the corneal centre and 2 mm from the corneal centre nasally, temporally, superiorly, and inferiorly. Data on conjunctival surface temperature, 8 mm temporally, from the corneal centre were also collected (Figure 1). We chose 8 mm temporally from the corneal centre following a detailed examination of all collected images, as this was the location at which data could be extracted for all participants; the nasal, superior, and inferior conjunctival locations were covered by the eyelids in most of the participants.

Next, all participants underwent a complete standard ophthalmological examination. The best spectacle corrected visual acuity was determined. In addition, patients were examined using a TMS-5 corneal topographer (Tomey, Nürnberg, Germany) and a rotating Scheimpflug camera (Pentacam HR, Oculus Optikgeräte GmbH, Wetzlar, Germany). From the anterior corneal surface, we extracted the following parameters: the surface asymmetry index (SAI), surface regularity index (SRI), Klyce/Maeda keratoconus index (KCI), Smolek/Klyce keratoconus severity index (KSI), and keratoconus prediction index (KPI). From the tomographic examination, the following parameters were extracted: index of surface variance (ISV), index of surface asymmetry (IVA), keratoconus index (KI), central keratoconus index (CKI), index of height asymmetry (IHA), index of height decentration (IHD), central corneal thickness (CCT), pachymetry at the centre of the pupil (PCP), and pachymetry at the corneal apex (PCA).

A total of 179 eyes in 90 patients with KC (topographic KC classification (TKC) 0-1 to 4) and 82 eyes in 41 controls were examined. Participants' age at the time of examination was 36.1 ± 12.5 (range 14–67) years in the KC group and 36.4 ± 12.8 (range 18–78) years in the control group (*p*=0.923). The KC group was 34.1% females and included 53.6% left eyes, whereas the control group was 52.4% females and included 47.6% left eyes. Thirty-one (38%) eyes in the control group (71% soft and 29% rigid contact lenses) and 78 (44%) eyes (all rigid contact lenses) in the KC group had previous contact lens wear. Unfortunately, we could not gather information on the number of hours with occasional/daily contact lens wear.

Statistical analyses were performed using SPSS software (SPSS version 19.0, IBM, New York). For the statistical analysis, both eyes were used for each participant based on the assumption that each eye and its measurements was an independent sample. Nevertheless, we have to take into account that the participants cannot discriminate between a worse and a better eye, as the questionnaire summarizes the symptoms for both eyes. Therefore, analysing the correlation of the OSDI score/subscores is always biased through analysis of the other eye in the same patient.

A test for normal distribution was performed qualitatively for both groups using the P-P plot. The Mann–Whitney *U* test was then used to investigate differences in SAI, SRI, KCI, KSI, KPI, ISV, IVA, KI, CKI, IHA, IHD, OSDI scores, vision- and discomfort-related OSDI subscores, and central,/superior/inferior/nasal/conjunctival OST, CCT, PCP, and PCA between both groups. *P* values less than 0.05 were considered significant.

The Spearman correlation test was used to analyse the interaction between OSDI, vision- and discomfort-related OSDI subscores, SAI, SRI, and OST  at the corneal centre and other corneal and conjunctival regions, and CCT (all are metric variables). The Spearman correlation was also used to analyse the interaction between OST  and age, the indices SAI and SRI, and CCT. The strength of the correlation was determined to be very strong (*r* ≥ 0.8), moderately strong from 0.6 to 0.8, fair from 0.3 to 0.5, and poor <0.3 [32].

## 3. Results

Best spectacle corrected visual acuity was 0.6 ± 0.3 in the KC group and 0.9 ± 0.2 in controls. The refractive cylinder was −3.5 ± 2.8 D in the KC group and −1.0 ± 1.0 D in controls. From corneal topography and tomography, SAI, SRI, KCI, KSI, KPI, ISV, IVA, KI, CKI, IHA, and IHD are given in Table 1 for both groups. Tables 2–3 provide the OSDI scores and subscores, corneal and conjunctival OST values, and CCT, PCP, and PCA in both groups. We found a significant difference in SAI, SRI, KCI, KSI, KPI, ISV, IVA, KI, CKI, IHA, IHD, CCT, PCP, and PCA between both groups (*p* < 0.001).

The OSDI score (31.4 ± 22.4 vs. 17.5 ± 17.9), vision-related subscore (17.7 ± 14.6 vs. 10.5 ± 13.2), and discomfort-related subscore (14.3 ± 10.7 vs. 9.4 ± 10.5) were significantly higher in the KC patients than in controls (*p* < 0.001). The average central OST was 34.2 ± 0.6°C in KC patients and 34.2 ± 0.6°C in controls (*p*=0.56). We found no significant difference in central (34.2 ± 0.6°C vs. 34.2 ± 0.7°C), nasal (34.2 ± 0.6°C vs. 34.2 ± 0.7°C), temporal (34.2 ± 0.6°C vs. 34.2 ± 0.6°C), and superior (34.2 ± 0.6°C vs. 34.2 ± 0.6°C) OST between the two groups (*p* ≥ 0.22).

According to TKC, 24 eyes were classified as stage 1 (13.4%), 55 eyes as stage 2 (30.7%), 51 eyes as stage 3 (28.5%), and 24 eyes as stage 4 (13.4%). Patients with a TKC between two stages (e.g., TKC 0-1) were always classified as the more advanced stage. Using the Kruskal–Wallis test, we found no difference in the OST between less and more advanced stages of KC; therefore, we did not perform a correlation analysis for the KC subgroups.

OSDI score poorly correlated with the SAI (*r* = 0.295, *p* < 0.001) and fairly correlated with the SRI (*r* = 0.354, *p* < 0.001), but did not correlate with OST at the corneal centre (*r* = −0.012) or other corneal or conjunctival regions (*r* ≥ −0.072). OSDI also did not correlate with CCT in either group (*r* = −0.270).

The correlation of the vision- and discomfort-related OSDI subscores with SAI, SRI, and OST  at the corneal centre in different stages of KC are shown in Table 4. For all participants, vision- and discomfort-related OSDI subscores poorly to fairly correlated with SRI and SAI (*r* > 0.174, *p* < 0.005), but none of the subscores correlated with OST (*r* < 0.001). In some of the subgroups (control, KC1, and KC2), the subscores correlated poorly with SAI and SRI, and the discomfort-related OSDI subscore poorly correlated with OST (Table 4).

OST at all of the examined regions also fairly correlated with patient age (−0.177 ≥ *r* ≥ −0.310) in the KC group and did not correlate with the control group (−0.10 ≥ *r* ≥ −0.074). OST  at the corneal centre also did not correlate with the SAI (*r* = −0.056), SRI (*r* = −0.086), or CCT (*r* = 0.048).

## 4. Discussion

The most conspicuous finding of our study is that OST does not differ between KC patients and controls, though the OSDI score was significantly higher in KC patients than in controls. In addition, OST at the corneal centre did not correlate with SAI, SRI, TKC (*p* ≥ 0.18), CCT, PCP, or PCA (*p* ≥ 0.06). The OSDI score and vision- and discomfort-related OSDI subscores poorly to fairly correlated with the SAI and SRI, but did not correlate with central corneal OST.

The aetiology of KC remains unclear. However, several authors have discussed a potential inflammatory cofactor [22–24]. Allergic conjunctivitis and dry eye syndrome are very common among KC patients [21, 26]. In patients with dry eye, the OST is increased, and OST decreases quicker during sustained eye opening than in healthy adults [7, 32–34]. Moussa et al. [35] could not find any diurnal changes in the OST of healthy adults. Morgan et al. [8] also found an increase in the OST throughout the day, especially in dry eyes. These findings suggest that diurnal changes in OST indicate ocular surface abnormalities or corneal pathology. Analysing the diurnal changes in the OST of KC patients was not the aim of the present study, but it could be interesting to assess the diurnal variations in OST in KC patients in the future.

Hara et al. found a significant correlation between the conjunctival surface temperature and the severity of conjunctival allergic disease, and OST was a useful measure to determine the effectiveness of antiallergy agents [36]. The increase in the OSDI score in KC patients may reflect dry eye disease or could be related to the poor visual outcomes in KC patients. With an increase in both the vision- and discomfort-related OSDI subscores in KC, we could determine that both ocular surface disease and deteriorated visual acuity contribute to the increased OSDI score. However, this is not mirrored by an increase in the OST. Data on conjunctival allergic disease were not collected in the present study.

Corneal innervation may also play a decisive role in OST. The cornea is densely innervated by the fibres of the ophthalmic branch of the trigeminus nerve, known as ciliary nerves [37]. Corneal nerves are important for corneal homeostasis due to their protective functions and their role in wound healing and in regulating corneal sensation [38, 39]. Epithelial dendritic cells (Langerhans cells) are inflammatory, antigen-presenting corneal cells responsible for immune surveillance. These cells are distributed from the basal epithelial corneal layer to the sub-basal nerve plexus [40]. Mature Langerhans cell morphology is frequently seen in the periphery of the cornea, whereas immature cells are seen centrally [41].

The sub-basal nerve plexus and the epithelial dendritic cell density have been examined in different subtypes of dry eye disease. Tepelus et al. [42] found a reduction in the sub-basal nerve plexus and an increase in inflammatory dendritic cell density in Sjögren and non-Sjögren dry eye subgroups [43]. Many studies have demonstrated abnormal corneal nerve morphology and branching patterns, reduced nerve density, increased tortuosity, and thickening in KC [44, 45]. Mandathara et al. found mature Langerhans cells at the centre of the cornea, which also supports an inflammatory origin of KC [46]. To the best of our knowledge, the relationship between changes in the sub-basal nerve plexus and Langerhans cell density and OST has not yet been analysed.

The literature offers controversial information on the effect of corneal thickness on OST. Morgan reported a significant decrease in OST with increasing corneal thickness [47]. In contrast, Alio and Padron [48] and Efron et al. [49] found a progressive increase in OST from the corneal centre to the periphery. Purslow and Wolffsohn [50] found a weak negative correlation between corneal thickness and OST using the Thermo Tracer 7210MX. Pattmöller et al. [51] could not verify a correlation between local corneal thickness and local OST at any point on the corneal surface in healthy adults. The relation of the OST and the anterior chamber depth is also contraversial [48, 49, 51]. In the present study, we could not determine a correlation between corneal thickness and OST in KC patients or controls.

In summary, we found a significantly increased OSDI score in KC patients compared to an age-matched control group. However, this was not accompanied by an increase in the OST  at any stage of KC. We could not clarify whether the reduced corneal thickness in KC patients may have a “corneal-cooling effect”. Our study also shows that both vision- and discomfort-related symptoms of KC have to be managed in parallel in ophthalmologic practice, but the necessity of anti-inflammatory treatment cannot be verified through ocular thermography.

## Figures and Tables

**Table 1:** Corneal topographic and tomographic data for the keratoconus patients and controls.

	SAI	SRI	KCI	KSI	KPI	ISV	IVA	KI	CKI	IHA	IHD
Keratoconus	2.1 ± 1.6	1.0 ± 0.6	57.0 ± 37.2	50.3 ± 29.4	0.35 ± 0.1	84.7 ± 49.4	0.9 ± 0.5	1.2 ± 0.2	1.1 ± 0.1	26.5 ± 22.2	0.1 ± 0.1
Control	0.4 ± 0.2	0.2 ± 0.2	1.8 ± 6.4	2.5 ± 8.3	0.2 ± 0.02	18.16 ± 7.6	0.1 ± 0.1	1.0 ± 0.2	1.0 ± 0.0	6.0 ± 5.5	0.01 ± 0.01
*p* value	**<0.01**	**<0.01**	**<0.01**	**<0.01**	**<0.01**	**<0.01**	**<0.01**	**<0.01**	**<0.01**	**<0.01**	**<0.01**

Data are given as mean ± SD. SAI, surface asymmetry index; SRI, surface regularity index; KCI, Klyce/Maeda keratoconus index; KSI, Smolek/Klyce neural network index; KPI, keratoconus prediction index; ISV, index of surface variance; IVA, index of vertical asymmetry; KI, keratoconus index; CKI, central keratoconus index; IHA, index of height asymmetry; IHD, index of height decentration. A significant difference was found in all factors between both groups (*p* < 0.01).

**Table 2:** Ocular Surface Disease Index (OSDI) score and subscores, ocular surface temperature (OST), and corneal thickness at different regions.

	OSDI	VR OSDI subscore	DR OSDI subscore	Central OST	Superior OST	Inferior OST	Nasal OST	Temporal OST	Conjunctival OST	CCT	Pachymetry at the pupil	Pachymetry at the apex
Keratoconus	31.5 ± 22.0	17.7 ± 14.6	14.3 ± 10.7	34.3 ± 0.6	34.2 ± 0.6	34.2 ± 0.7	34.2 ± 0.6	34.2 ± 0.6	34.5 ± 1,0	475.0 ± 50.4	489.6 ± 45.5	480.1 ± 51.5
Control	17.5 ± 17.5	10.5 ± 13.2	9.4 ± 10.5	34.3 ± 0.7	34.2 ± 0.7	34.7 ± 0.6	34.2 ± 0.7	34.2 ± 0.6	34.6 ± 0.8	529.1 ± 35.2	545.6 ± 30.8	546.8 ± 30.7
*p* value	**<0.001**	**<0.001**	**<0.001**	0.414	0.273	0.221	0.361	0.283	0.283	**<0.001**	**<0.001**	**<0.001**

Data are given as mean ± SD. CCT, central corneal thickness; VR, vision-related, DR, discomfort-related.

**Table 3:** Ocular Surface Disease Index (OSDI) score and subscores and central and conjunctival ocular surface temperature (OST) in different stages of keratoconus (KC) and controls.

	OSDI	VR subscore	DR subscore	Central OST	Conjunctival OST
KC4	41.9 ± 25.9	25.6 ± 18.0	17.5 ± 12.5	34.2 ± 0.6	34.5 ± 0.9
KC3	29.3 ± 18.5	16.3 ± 11.9	13.0 ± 9.7	34.3 ± 0.7	34.5 ± 1.2
KC2	31.7 ± 25.1	17.6 ± 15.9	14.8 ± 12.4	34.2 ± 0.6	34.5 ± 0.9
KC1	30.1 ± 19.2	15.4 ± 11.5	14.0 ± 8.9	34.3 ± 0.7	34.3 ± 1.0
Control	17.5 ± 17.5	10.5 ± 13.2	9.4 ± 10.5	34.3 ± 0.7	34.6 ± 0.8

Data are given as mean ± SD. VR, vision-related; DR, discomfort-related.

**Table 4:** Spearman correlation of vision- and discomfort-related Ocular Surface Disease Index (OSDI) subscores with surface characteristics at the corneal centre in different stages of keratoconus (KC) and controls.

	SAI	SRI	Central OST
KC4–VR OSDI	−0.29	−0.08	−0.18
KC3–VR OSDI	−0.12	−0.05	−0.007
KC2–VR OSDI	−0.07	0.26 *p*=0.04	0.03
KC1–VR OSDI	0.33	0.02	0.35
Control–VR OSDI	0.22 *p*=0.03	0.32 *p*=0.002	−0.07
KC4–DR OSDI	−0.18	0.24	−0.09
KC3–DR OSDI	−0.27	−0.19	−0.04
KC2–DR OSDI	−0.15	0.18	0.02
KC1–DR OSDI	0.27	0.18	0.45 *p*=0.02
Control–DR OSDI	0.26 *p*=0.01	0.25 *p*=0.01	−0.01

VR, vision-related; DR, discomfort-related; SAI, surface asymmetry index; SRI, surface regularity index; OST, ocular surface temperature. *r* values are given, with *p* values shown in the case of significance.

## Data Availability

The data used to support the findings of this study are included within the article.
